# Hyperoside Attenuates Sepsis-Induced Acute Lung Injury (ALI) through Autophagy Regulation and Inflammation Suppression

**DOI:** 10.1155/2023/1257615

**Published:** 2023-07-27

**Authors:** Jingyin Mai, Qingqing He, Yuting Liu, Yuting Hou

**Affiliations:** ^1^Emergency Department, Shanghai Municipal Hospital of Traditional Chinese Medicine, Shanghai University of Traditional Chinese Medicine, Shanghai, Shanghai 200071, China; ^2^Hospital Infection Management Department, Guanghua Hospital Affiliated to Shanghai University of Traditional Chinese Medicine, Shanghai, Shanghai 200052, China; ^3^Cardiovascular Department, Guanghua Hospital Affiliated to Shanghai University of Traditional Chinese Medicine, Shanghai, Shanghai 200052, China; ^4^Department of Pharmacy, Guanghua Hospital Affiliated to Shanghai University of Traditional Chinese Medicine, Shanghai, Shanghai 200052, China

## Abstract

**Background:**

Sepsis mortality and morbidity are aggravated by acute lung injury (ALI) or acute respiratory distress syndrome. Published studies have discovered that hyperoside (HYP) has an anti-inflammatory and therapeutic effect in many diseases. However, whether HYP treatment can attenuate sepsis-induced ALI is still obscure.

**Methods:**

In this study, a cecal ligation and puncture (CLP)-induced sepsis mouse model was constructed. The mouse lungs were harvested and assessed using proteomics, immunohistochemistry, immunofluorescence, and enzyme-linked immunosorbent assay for pro-inflammatory cytokines. Human lung microvascular endothelial cells (HLMVECs) were induced with lipopolysaccharide (LPS) for the *in vitro* model.

**Results:**

The results showed that HYP treatment attenuated sepsis-induced ALI through an increased survival rate, decreased inflammatory factor expression, and lung tissue apoptosis. At the same time, HYP pretreatment restored angiogenesis in CLP-induced mouse lung tissues. Proteomics detection showed that Atg13 played a vital role in HYP-mediated protection against sepsis-induced ALI. The *in vitro* experiment showed HYP treatment attenuated LPS-induced HLMVEC damage by regulating Atg13-mediated autophagy. Inhibiting autophagy or silencing Atg13 reversed the protective effect of HYP against sepsis-induced ALI.

**Conclusion:**

Taken together, we conclude that HYP attenuated sepsis-induced ALI by regulating autophagy and inhibiting inflammation.

## 1. Introduction

Sepsis is a major health burden worldwide because it is one of the most unaffordable conditions of hospitalization and also because it is the leading cause of mortality worldwide [1, 2]. It has become one of the main clinical predisposing factors for acute lung injury (ALI), a syndrome including multiple acute respiratory failure diseases [3, 4]. In the progression of ALI induced by sepsis, the upregulation of inflammatory and apoptotic pathways destroys alveolar epithelial cells, increases epithelial permeability, and leads to the influx of edema fluid into the alveolar space [5, 6]. Many studies found that the inflammatory factor expression disrupted vascular integrity, indicating its involvement in vascular injury [7, 8]. Nevertheless, its role and mechanism of action in inflammatory lung injury have not been elucidated.

Hyperoside (HYP) (quercetin 3-*o*-*β*-d-galactopyranoside) is a flavonoid glycoside extracted from *Rhododendron brachycarpum* with anti-inflammatory effects [9, 10]. Previous studies found that HYP alleviated allergic airway inflammation via nuclear factor erythroid-related factor 2 (Nrf2) pathway activation [11, 12]. However, whether HYP can attenuate sepsis-induced lung vascular injury is still unclear.

In this regard, we discovered that HYP treatment attenuated sepsis-induced ALI. The mechanistic study discovered that HYP treatment restored the function of vascular endothelial cells by regulating Atg13-mediated autophagy. This research indicated the treatment efficacy of HYP and the mechanism in sepsis-induced ALI via Atg13-mediated autophagy.

## 2. Materials and Methods

### 2.1. Sepsis Animal Model

The experimental cecal ligation and puncture (CLP) mouse model of sepsis was established, as described previously [13]. Briefly, BALB/c mice were anesthetized by isoflurane inhalation, and then the stump was punctured once with a 22-gauge needle to squeeze out a tiny amount of feces. The cecum was placed back to the normal intra-abdominal position, and the abdomen was closed. After that, 0.3 mL of saline was administrated subcutaneously for fluid resuscitation. Sham-operated control mice underwent the same surgical procedure without ligation or puncture. The mice were placed back into their cages and provided with food and water *ad libitum*. We monitored mortality daily for 7 days. The mice were intraperitoneally administered HYP at a concentration of 40 mg/kg body weight 1 hr before CLP to examine the effect of HYP. For autophagy regulation, 3-methyladenine (3-MA), an autophagy inhibitor, was intraperitoneally administered at a concentration of 15 mg/kg body weight 1 hr before CLP, as previously mentioned [14].

### 2.2. Cell Culture

Human lung microvascular endothelial cells (HLMVECs) (Cell Applications Inc., CA, USA) were cultured in EBM-2 (containing endothelial growth factor, CC3162, Lonza, MD, USA) supplemented with 5% FBS (Gibco, CA, USA) at 37°C, 5% CO_2_, and 95% humidity and passaged every 3–5 days. P4–P7 cells were utilized in the next stage of the experiment. Lipopolysaccharide (LPS) (Sigma-Aldrich, MO, USA; 10 *μ*g/mL) treatment for 1 day was applied on HLMVECs for the *in vitro* model. Then, 50 *μ*M HYP or 3 mM 3-MA was used to investigate the regulatory role of HYP on autophagy. For Atg13 silencing (si-Atg13 : 5′-AAGUCCCUUCUUGCUAUAACUAGTTCUAGUUAUAGCAAGAAGGGACUUTT-3′), siRNA against Atg13 was used to transfect into HLMVECs using Lipofectamine 2000 reagent (Invitrogen, Carlsbad, CA, USA). After transfection for 48 hr, HLMVECs cells were harvested for use in subsequent experiments.

### 2.3. Enzyme-linked Immunosorbent Assay (ELISA) for Soluble Inflammatory Cytokines

The expression of inflammatory factors IL-6, IL-1*β*, and TNF-*α* in serum or HLMVEC cell supernatants were detected utilizing ELISA kits (Sen-Xiong Company, Shanghai, China). Following the protocols, the supernatants were maintained at –80°C prior to the measurement, and the standards and samples were run in triplicate. OD_450_ was computed by subtracting the background, and standard curves were drawn.

### 2.4. Cell Apoptosis Assay

An Annexin *V*/propidium iodide (PI) staining kit (Abcam, MA, USA) was employed for apoptosis assay. Cells (1 × 10^3^/well) were seeded in 96-well plates for 24 hr. Thereafter, the cells were trypsinized, washed with phosphate-buffered saline, resuspended in binding buffer, and stained with Annexin V–fluorescein isothiocyanate and PI for 15 min at room temperature in the dark. The apoptotic rate was determined by a NovoCyte 2000 Flow Cytometer (Agilent, CA, USA).

### 2.5. RNA Isolation and Real-Time Polymerase Chain Reaction (PCR)

Total RNA was obtained with TRIzol Reagent (Invitrogen, CA, USA), followed by cDNA synthesis using TransScript All-in-One First-Strand cDNA Synthesis SuperMix (Transgen Biotech, Beijing, China). PCR was performed using a Bio-Rad PCR instrument (Bio-Rad, CA, USA) with 2x Taq PCR Master Mix (Solarbio, Beijing, China) following the manufacturer's protocols. The fold changes were computed with relative quantification means by the 2^−*△△*Ct^ approach. PCR primers are attached in Table 1.

### 2.6. Tubule Formation Assay

*In vitro* neovascularization was measured in fibrin matrices. After treatments, the serum-starved HLMVECs in the endothelial basal medium were seeded onto Matrigel-coated plates (10^5^ cells/well into six wells) (BD Biosciences, NJ, USA) and incubated at 37°C for 12 hr. The tubular structures formed in Matrigel were observed and photographed by phase contrast microscopy, and the lengths of the newly formed tubes in 10 randomly selected fields per well were measured.

### 2.7. Immunohistochemistry and Immunofluorescence Analysis

The lung tissue samples were fixed in 10% formalin solution, embedded in paraffin, and sectioned at 5 *μ*m. Sections were stained with terminal deoxynucleotidyl transferase dUTP nick end labeling (TUNEL) for histological evaluations. The section was examined using an Axiophot light microscope (Zeiss, Oberkochen, Germany). For CD31 detection, sections were fixed in a 10% formalin solution and stained with CD31. The sections were examined with a fluorescence microscope (Nikon, Tokyo, Japan) and photographed with a digital camera.

### 2.8. Statistical Analysis

The continuous variables were denoted by means ± standard deviation. We used one-way analyses of variance for comparisons using GraphPad Prism (GraphPad, CA, USA). A *P* value ≤ 0.05 indicated a statistically significant difference.

## 3. Results

### 3.1. HYP Treatment Attenuated Sepsis-Induced ALI

**Figure 1:**
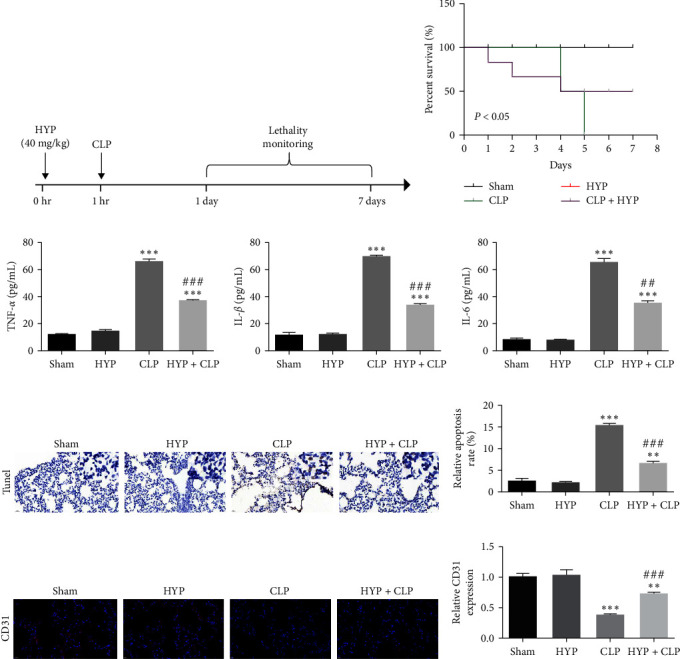
Hyperoside (HYP) treatment attenuated sepsis-induced ALI. (a) schematic design of experimental procedures. Mice (*n* = 12, each group). (b) Survival of control and HYP-treated mice with sepsis was tracked for 7 days. (c–e) ELISA detection showcased the expression of inflammatory factors TNF-*α*, IL-1*β*, and IL-6. (f and g) Immunohistochemical analysis for TUNEL detection showed apoptosis of lung tissue. (h and i) Immunofluorescence for CD31 staining showed angiogenesis in lung tissue. Data were denoted as means ± SD.  ^*∗∗*^*P* < 0.01,  ^*∗∗∗*^*P* < 0.001 vs. the sham group. ^###^*P* < 0.001 vs. the CLP group.

We monitored mouse survival after CLP with or without HYP (40 mg/kg) for 7 days to examine HYP effects upon septic mortality (Figure 1(a)). According to Kaplan–Meier survival curves, the result showed that mice with CLP-induced sepsis had no survival after 7 days. However, HYP pretreatment improved the survival rate (Figure 1(b)). ELISA detection (3 days after CLP) revealed that the expression of inflammatory factors IL-1*β*, TNF-*α*, and IL-6 incremented significantly in the CLP group. Nevertheless, HYP pretreatment decreased the CLP-induced expression of inflammatory factors (Figure 1(c)–1(e)). The immunohistochemical analysis for TUNEL detection showed that the apoptosis in lung tissues increased in mice with CLP-induced sepsis. However, the apoptotic rate decreased in the HYP pretreatment group (Figures 1(f) and 1(g)). Immunofluorescence for CD31 staining (3 days after CLP) showed that the microvascular endothelial cells in lung tissues were destroyed in mice with CLP-induced sepsis. However, angiogenesis increased in the HYP pretreatment group (Figures 1(h) and 1(i)).

### 3.2. Atg13 Played an Important Role in HYP-Mediated Protection against Sepsis-Induced ALI

**Figure 2:**
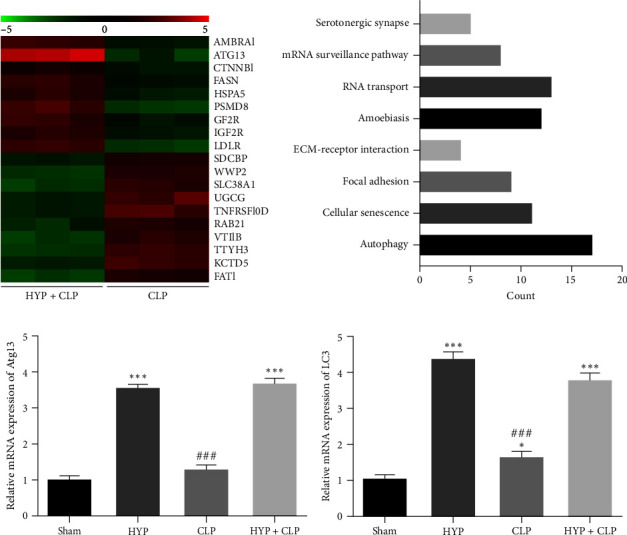
Atg13 played an important role in HYP-mediated protection against sepsis-induced ALI. (a) Proteomics detection with heat map analysis showed the abnormal expression of the protein in lung tissues from normal (NC) and sepsis (CLP) mice. (b) KEGG pathway enrichment analyses. (c and d) RT-qPCR detection showcased the expression of Atg13 and LC3 in lung tissues. Data are denoted as means ± SD.  ^*∗∗*^*P* < 0.01,  ^*∗*^*P* < 0.05,  ^*∗∗∗*^*P* < 0.001 vs. the sham group. ^###^*P* < 0.001 vs. the HYP group.

The proteomic analysis showed that HYP treatment resulted in the abnormal expression of proteins in lung tissues in mice with sepsis-induced ALI, including Atg13 expression in the HYP treatment group (Figure 2(a)). KEGG pathway enrichment analysis also discovered that the autophagy pathways were enriched (Figure 2(b)). RT-qPCR detection showed that HYP treatment increased Atg13 and LC3 expression (Figures 2(c) and 2(d)). This suggested that Atg13 played an important role in HYP-mediated protection against sepsis-induced ALI by activating autophagy.

### 3.3. HYP Treatment Attenuated LPS-Induced HLMVEC Damage by Regulating Atg13-Mediated Autophagy

**Figure 3:**
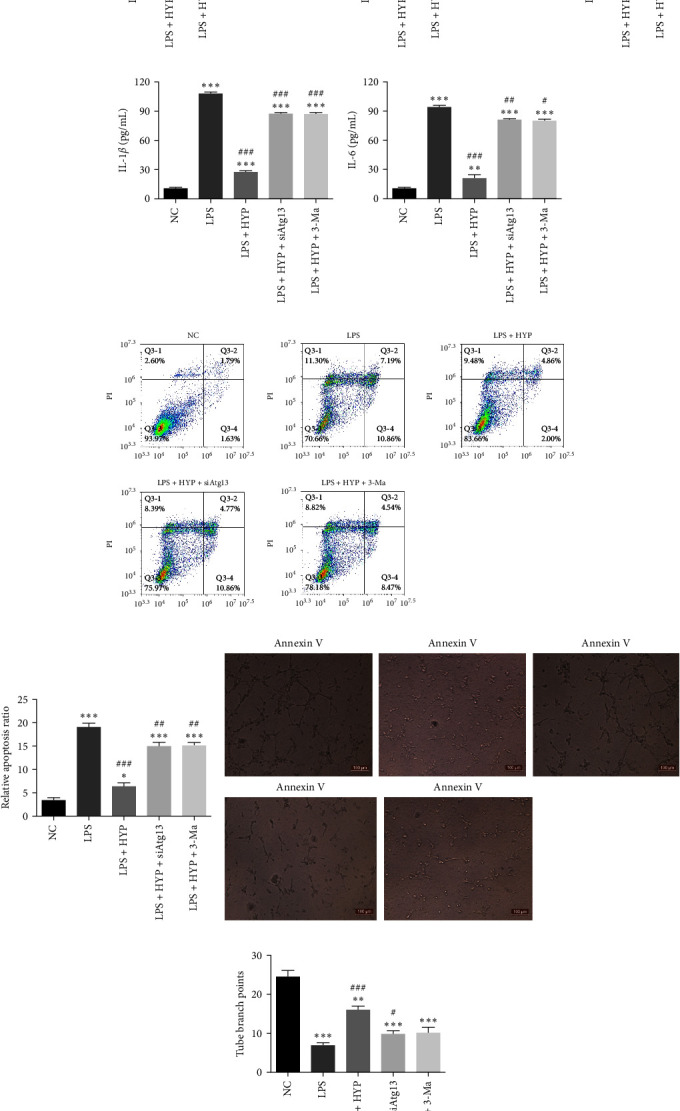
HYP treatment attenuated LPS-induced HLMVEC damage by regulating Atg13-mediated autophagy. (a and b) RT-qPCR detection showcased the expression of Atg13 and LC3 in HLMVEC cells. (c–e) ELISA detection showed the expression of inflammatory factors TNF-*α*, IL-1*β*, and IL-6. (f and g) HLMVEC cells apoptosis was assayed via flow cytometry after Annexin V–FITC staining. (h and i) *In vitro* tube formation of HLMVECs. The total branching point was analyzed. Data are denoted as means ± SD.  ^*∗*^*P* < 0.05,  ^*∗∗*^*P* < 0.01,  ^*∗∗∗*^*P* < 0.001 vs. the NC group. ^#^*P* < 0.05, ^##^*P* < 0.01, ^###^*P* < 0.001 vs. the LPS group.

HLMVECs were used for the *in vitro* model with 10 *μ*g/mL LPS treatment for 1 day to reveal whether Atg13-mediated autophagy played an important role. The result of RT-qPCR detection showed that HYP treatment increased Atg13 and LC3 expression based on the induction by LPS (Figures 3(a) and 3(b)). However, the silencing of Atg13 decreased the expression of both Atg13 and LC3. Autophagy inhibitor 3-MA treatment significantly decreased LC3 expression but could not inhibit Atg13 expression. This suggested that Atg13 played an important role in HYP-mediated protection over sepsis-induced ALI by activating autophagy.

ELISA detection inferred that the expression of inflammatory factors TNF-*α*, IL-1*β*, and IL-6 in HLMVEC cultivation incremented significantly in the LPS-induced group. However, HYP treatment inhibited LPS-induced inflammatory factor expression. Atg13 downregulation or autophagy inhibition restored inflammatory factor expression (Figure 3(c)–3(e)).

Flow cytometric analyses with Annexin V/PI staining revealed that HYP treatment inhibited LPS-induced HLMVEC apoptosis. The downregulation of Atg13 or inhibition of autophagy restored the HLMVEC apoptosis (Figures 3(f) and 3(g)). The *in vitro* tube formation of HLMVECs showed that LPS treatment decreased the ability of angiogenic differentiation. HYP treatment restored angiogenic differentiation ability under LPS-induced conditions. However, the downregulation of Atg13 or inhibition of autophagy reversed the protective effect of HYP on HLMVEC function under LPS conditions (Figures 3(h) and 3(i)).

### 3.4. Inhibition of Autophagy Reversed the Protective Effect of HYP against Sepsis-Induced ALI

**Figure 4:**
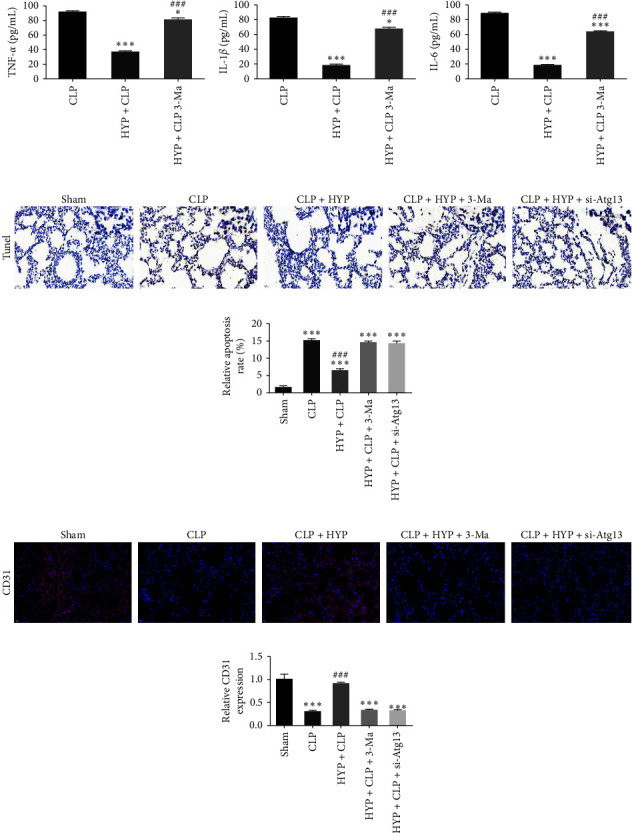
Autophagy inhibition reversed the protective effect of HYP against sepsis-induced ALI. (a–c) ELISA detection showcased the expression of inflammatory factors TNF-*α*, IL-1*β*, and IL-6. Data are denoted as means ± SD.  ^*∗*^*P* < 0.05,  ^*∗∗∗*^*P* < 0.001 vs. the CLP group. ^###^*P* < 0.001 vs. the HYP + CLP group. (d and e) Immunohistochemical analysis for TUNEL data showcased apoptosis of lung tissue. (f and g) Immunofluorescence for CD31 staining showed angiogenesis in lung tissue. Data are denoted as means ± SD.  ^*∗∗∗*^*P* < 0.001 vs. the sham group. ^###^*P* < 0.001 vs. the CLP group.

ELISA detection inferred that the expression of inflammatory factors IL-1*β*, TNF-*α*, and IL-6 in serum decreased significantly in the CLP group after HYP pretreatment. However, 3-MA treatment restored CLP-induced expression of inflammatory factors (Figure 4(a)–4(c)). Immunohistochemical analysis for TUNEL detection shows that the apoptosis in lung tissues decreased in mice with CLP-induced sepsis after HYP pretreatment. However, apoptosis was restored in the 3-MA treatment group (Figures 4(d) and 4(e)). Immunofluorescence for CD31 staining showed that the microvascular endothelial cells in lung tissues were restored in mice with CLP-induced sepsis after HYP pretreatment. However, angiogenesis decreased in the 3-MA treatment group (Figures 4(f) and 4(g)).

## 4. Discussion

Sepsis is a common and potentially fatal systemic disorder that generally results in uncontrollable inflammation, tissue damage, and multiple-organ failure. Sepsis and its complications are the leading causes of mortality among intensive care unit patients [15, 16]. Excessive inflammation and severe lung epithelial cell apoptosis are regarded as potential mechanisms of sepsis-induced ALI [17, 18]. In our research, we discovered that HYP treatment attenuated sepsis-induced ALI by increasing the survival of mice. The study also found that HYP treatment inhibited the expression of inflammatory cytokines and lung epithelial cell apoptosis. Previous studies also confirmed that HYP had an anti-inflammatory effect [19, 20].

In this study, the proteomics detection showed that Atg13 expression increased in the lung tissues of mice in the HYP treatment group. In the present study, we systematically investigated the individual importance of ATG13 interaction sites for ULK1 complex formation, recruitment to the autophagosome formation site, and autophagy induction [21, 22]. The data of this study also found that the downregulation of Atg13 inhibited autophagy-related protein LC3 expression. 3-MA treatment had a similar effect as Atg13 silencing, which reversed the protective effects of HYP against sepsis-induced ALI. *In vitro* experiments confirmed that HYP treatment inhibited HLMVEC damage by regulating Atg13-mediated autophagy. The result also found that HYP treatment inhibited LPS-induced inflammatory factor expression and HLMVEC apoptosis. At the same time, HYP treatment restored the tube formation ability of HLMVECs. The *in vivo* experiment further confirmed that HYP treatment inhibited sepsis-induced ALI by autophagy activation. The autophagy inhibition reversed the protective effects of HYP against sepsis-induced ALI. Previous studies also showed that HYP attenuated pregnancy loss by activating autophagy and suppressing inflammation in a rat model [23]. The study also found that sepsis is a leading cause of maternal morbidity, with a high case fatality rate and leads to significant perinatal loss. The physiological changes of pregnancy and puerperium make pregnant women more susceptible to sepsis and also pose a challenge for early diagnosis because of the overlap of clinical features and laboratory values [24]. Systemic inflammation, as measured by high-sensitivity C-reactive protein, has also been hypothesized to play a role in infertility and perinatal loss [25]. So, inhibit inflammation mediated by sepsis has an important clinical significance.

Previous studies have confirmed HYP attenuated inflammation in HT22 cells via upregulating SIRT1 to activities Wnt/*β*-Catenin and sonic hedgehog pathways [10]. The study also found that HYP attenuates OVA-induced allergic airway inflammation by activating Nrf2 [12]. But the regulatory mechanism of HYP-attenuated sepsis-induced inflammation is still unclear and needs further clarification.

In summary, we suggested that HYP attenuated sepsis-induced ALI via regulating Atg13-mediated autophagy and inhibiting inflammation. However, the specific regulatory mechanism needs to be further validated. The findings of the present study might help speculate on the therapeutic value of HYP.

## Figures and Tables

**Table 1:** The primer for different gene.

Gene	Forward (5′⟶3′)	Reverse (5′⟶3′)
Atg-13 (human)	TAAAGATGACATTCTTCCGATGGA	TTCTCATGCACAGCCAGCTT
Atg-13 (mice)	CCAGGCTCGACTTGGAGAAAA	AGATTTCCACACACATAGATCGC
LC3 (human)	GGTTTCCCGTCACCAATTTTCC	TGTGGTTTCCAACGTAGAGGA
LC3 (mice)	GGCTACGGCTACTATCGCAC	GCTGTCACCTTCACCGTTCC
*β*-actin (human)	CTCCATCCTGGCCTCGCTGT	GCTGTCACCTTCACCGTTCC
*β*-actin (mice)	GTGACGTTGACATCCGTAAAGA	GCCGGACTCATCGTACTCC

## Data Availability

The datasets generated and analyzed in the present study are available from the corresponding author upon reasonable request.
